# Diagnostic Accuracy Assessment of Immunochromatographic Tests for the Rapid Detection of Antibodies against *Orientia tsutsugamushi* Using Paired Acute and Convalescent Specimens

**DOI:** 10.4269/ajtmh.15-0435

**Published:** 2015-12-09

**Authors:** Wanitda Watthanaworawit, Paul Turner, Claudia Turner, Ampai Tanganuchitcharnchai, Suthatip Jintaworn, Borimas Hanboonkunupakarn, Allen L. Richards, Nicholas P. J. Day, Stuart D. Blacksell, François Nosten

**Affiliations:** Shoklo Malaria Research Unit, Mahidol Oxford Tropical Medicine Research Unit, Faculty of Tropical Medicine, Mahidol University, Tak, Thailand; Mahidol Oxford Tropical Medicine Research Unit, Faculty of Tropical Medicine, Mahidol University, Bangkok, Thailand; Centre for Tropical Medicine and Global Health, Nuffield Department of Medicine, University of Oxford, Oxford, United Kingdom; Department of Clinical Tropical Medicine, Faculty of Tropical Medicine, Mahidol University, Bangkok, Thailand; Viral and Rickettsial Diseases Department, Naval Medical Research Center, Silver Spring, Maryland

## Abstract

We assessed the diagnostic accuracy of two immunochromatographic tests (ICTs), the Access Bio *CareStart* Scrub Typhus test (Somerset, NJ) (IgM), and the SD BIOLINE Tsutsugamushi test (Kyonggi-do, Republic of Korea) (IgG, IgM, or IgA) compared with indirect immunofluorescence assay (IFA) and real-time PCR results as reference tests using 86 paired acute and convalescent specimens from febrile patients. The sensitivity and specificity of the *CareStart* test were 23.3% (95% confidence interval [CI]: 11.8–38.6) and 81.4% (95% CI: 66.6–91.6), respectively, for acute specimens and 32.6% (95% CI: 19.1–48.5) and 79.1% (95% CI: 64.0–90.0), respectively, for convalescent specimens. For the SD BIOLINE test, sensitivity and specificity were 20.9% (95% CI: 10.0–36.0) and 74.4% (95% CI: 58.8–86.5), respectively, for acute specimens and 76.7% (95% CI: 61.4–88.2) and 76.7% (95% CI: 61.4–88.2), respectively, for convalescent specimens. The poor sensitivity obtained for both ICTs during this study when performed on acute specimens highlights the difficulties in prompt diagnosis of scrub typhus.

## Introduction

Scrub typhus, caused by *Orientia tsutsugamushi*, is an important cause of acute undifferentiated febrile illness in Thailand^1^,^2^ and a common cause of fever on the Thailand–Myanmar border.^3^,^4^ Clinical manifestations are similar to other causes of fever such as dengue, leptospirosis, and malaria, making clinical diagnosis difficult.^4^ Laboratory confirmation relies on isolation of the organism at biosafety level 3 or paired serology (indirect immunofluorescence assay [IFA]) or on a combination of tests, that is, in vitro isolation, IFA, and polymerase chain reaction (PCR),^5^–^7^ which are expensive, time consuming, and do not provide results in time to inform patient management. Several groups have developed and assessed the clinical utility of rapid bedside diagnostic tests for early diagnosis of this infection to assist patient management.^8^–^11^ However, either diagnostic accuracy or commercial availability is limited. We evaluated the performance characteristics of two immunochromatographic tests (ICTs), the SD BIOLINE Tsutsugamushi (Standard Diagnostics, Inc., Kyonggi-do, Republic of Korea) test and the *CareStart* Scrub Typhus test (Access Bio, Inc., Somerset, NJ) using paired acute and convalescent serum specimens from patients with undifferentiated febrile illness.

## Materials and Methods

From a recent fever study in northwest Thailand, 86 participants were retrospectively selected for the current evaluation: 43 were confirmed to have acute scrub typhus infection and 43 patients were confirmed as not having acute scrub typhus infection by the detection of specific IgM antibody by IFA and real-time PCR assay targeting the *O. tsutsugamushi* 47-kDa outer membrane protein gene.^12^ Acute scrub typhus was defined as 1) ≥ 4-fold increase in IFA IgM titer, 2) seroconversion, 3) a high static titer of ≥ 1:25,600 between acute and convalescent specimens, and/or 4) PCR positive in the acute specimen. All specimens were stored at −80°C before testing. The study was approved by the Ethics Committee of the Faculty of Tropical Medicine, Mahidol University, Thailand (MUTM 2011-008-01) and Oxford Tropical Research Ethics Committee (OXTREC 42-10).

Two ICTs were assessed: the *CareStart* Scrub Typhus test for the detection of IgM antibody against *O. tsutsugamushi* (not currently commercially available) and the SD BIOLINE Tsutsugamushi test for the detection of IgG, IgM, or IgA antibodies to *O. tsutsugamushi*, which uses a recombinant *O. tsutsugamushi* (strains Kato, Karp, and Gilliam) surface antigen (available for purchase in Thailand for approximately 150 THB/US$ 4.6 /test and has the Conformité Européénne [CE] marking). Both ICTs were performed on both acute and convalescent specimens following the manufacturer's instructions. In brief, for the *CareStart* test, 10 μL serum was added to the test devices followed by one drop of assay buffer (40 μL). The results were read at 10 minutes. For the SD BIOLINE test, 10 μL serum was added to the test devices followed by three drops of assay diluent. The results of the tests were read at 15 minutes. Both tests had two lines, a test line “T” and a control line “C.” An absence of “C” line indicated an invalid result. The results of the tests were read by three independent readers, and the majority result was used for the final interpretation.

All statistical analyses were calculated using STATA/SE 10.1 (StataCorp., College Station, TX). Diagnostic accuracy of the tests was calculated by comparing the ICT results with the reference (composite IFA and PCR) results. A 2 × 2 table was constructed, in which the reference results were cross-tabulated with the ICT results to define the rate of true-positive, true-negative, false-positive, and false-negative results. The sensitivity, specificity, positive predictive value, and negative predictive value with 95% confidence intervals (CIs) were calculated using the “diagt” routine.^13^ Kappa values were generated to determine the level of interoperator variation in the reading of the ICT test results.^14^

## Results and Discussion

Of the 86 patients tested, 68.6% (59/86) were male. The median age was 20 years (interquartile range [IQR]: 14–35 years), median temperature at presentation was 38.6°C (IQR: 38.2–39.1°C), median duration of fever at the time of presentation was 2 days (IQR: 2–3 days), and the median interval between obtaining initial acute-phase specimens and convalescent specimens was 14 days (range: 11–30 days). The performance characteristics of the *CareStart* Scrub Typhus test and the SD BIOLINE Tsutsugamushi test compared with the results of the reference tests are shown in Table 1. The sensitivity of the *CareStart* test was low for both acute and convalescent specimens (23.3% [95% CI: 11.8–38.6] and 32.6% [95% CI: 19.1–48.5], respectively). The specificities of the *CareStart* test using both acute and convalescent specimens were 81.4% (95% CI: 66.6–91.6) and 79.1% (95% CI: 64.0–90.0), respectively. Both the sensitivity and specificity of the *CareStart* test were much lower in this study than in the study previously reported by Blacksell and others,^9^ which found the sensitivity to be 96.8% and the specificity to be 93.3% for acute-phase specimens. This may reflect the fact that our patients presented earlier in the course of their illness, when there is an absence of antibodies, with a median of 2 days (IQR: 2–3 days) of fever, compared with 8 days (range: 7–10 days) in the study of Blacksell and others. This result is consistent with the temporal antibody response for scrub typhus infectious.^15^

For the SD BIOLINE test, the sensitivity was higher for convalescent specimens (76.7% [95% CI: 61.4–88.2]) compared with acute specimens (20.9% [95% CI: 10.0–36.0]). The specificity of the test was similar when performed on acute and convalescent specimens (74.4% [95% CI: 58.8–86.5] and 76.7% [95% CI: 61.4–88.2], respectively). The sensitivity and specificity of the test obtained in this study were lower than those that the company claimed in the product insert (99% and 96%, respectively), although, the timing of blood collection was not mentioned. It is difficult to conclude, because of the limitations of the test, whether IgG, IgM, or IgA play a role in the positive result and whether the specificity of the test is compromised due to the detection of past infections.

Both ICTs tested in this study demonstrated very good interoperator agreement among three readers (Table 1). The SD BIOLINE test demonstrated better kappa values (0.98 and 0.98 for acute and convalescent specimens, respectively) compared with those of the *CareStart* test (0.84 and 0.85 for acute and convalescent specimens, respectively).

Given the small sample size of this evaluation, the 95% CI around the calculated sensitivities and specificities are wide. However, from these preliminary findings, both tests had poor sensitivity when performed on acute specimens taken early in the course of infection; however, this was improved for convalescent specimens. Consequently, both tests when performed at the acute phase of infection are not likely to be useful for patient management. Individual IgM or IgG antibody-based tests taken at appropriate time points post-onset of fever could be performed, which may give a clearer answer and aid the differentiation of past and current infections. In addition, there were four scrub typhus patients who had low IFA IgM titers on acute specimens (two were less than 100, one at 100, and one at 200, rising to 6,400, 12,800, 12,800, and 25,600 in convalescent specimens, respectively) but were positive by 47-kDa real-time PCR assay, none of whom were positive by either ICTs tested using acute specimens. This emphasizes the usefulness of nucleic acid detection during acute phase of scrub typhus infection. The eschar PCR could be useful for early diagnosis of scrub typhus. It is more likely to give a positive result than blood, especially, when patient has already been treated with antibiotics.^16^ In our study, blood specimens were collected before antibiotics were started, and none of the patients in this sample set presented with eschar at the enrollment. It should be noted that the use of IFA alone as the gold standard test has limitations, and it is far from a perfect gold standard as standard IFA slides are not available, there is no consensus on cutoff titers, and difficulties with cross-reactivity and subjective endpoint (reader variability). Therefore using it as the reference test could have affected negatively on the performance results of the novel tests.^6^,^17^ However, the appropriate gold standard for scrub typhus remains elusive.

This study illustrates the limitations of two ICTs for the timely diagnosis of scrub typhus, highlighting the need to further investigate the utility of other diagnostic methodologies, for example, antigen- or nucleic acid-based detection (which may be more useful for diagnosing scrub typhus, especially for patients presenting to the clinic early in the course of their illness, when antibody responses have not yet developed) and/or combination of antigen/nucleic acid with antibody-based rapid detection that may improve the diagnostic accuracy for early diagnosis.^5^,^18^

## Figures and Tables

**Table 1:** The performance characteristics of *CareStart* Scrub Typhus assay for detection of IgM antibody and SD BIOLINE Tsutsugamushi assay for detection of IgG, IgM, or IgA antibodies using acute and convalescent specimens compared with the results of reference tests*

Specimen timing and diagnosis	Positive	Negative	% Sensitivity (95% CI)	% Specificity (95% CI)	% PPV (95% CI)	% NPV (95% CI)	Kappa value
*CareStart* Scrub Typhus IgM (*N* = 86)							
Acute							
Acute scrub typhus	10	33	23.3 (11.8–38.6)	81.4 (66.6–91.6)	55.6 (30.8–78.5)	51.5 (39.0–63.8)	0.84
Not acute scrub typhus†	8	35					
Convalescent							
Acute scrub typhus	14	29	32.6 (19.1–48.5)	79.1 (64.0–90.0)	60.9 (38.5–80.3)	54.0 (40.9–66.6)	0.85
Not acute scrub typhus	9	34					
SD BIOLINE Tsutsugamushi IgG, IgM, or IgA (*N* = 86)							
Acute							
Acute scrub typhus	9	34	20.9 (10.0–36.0)	74.4 (58.8–86.5)	45.0 (23.1–68.5)	48.5 (36.0–61.1)	0.98
Not acute scrub typhus	11	32					
Convalescent							
Acute scrub typhus	33	10	76.7 (61.4–88.2)	76.7 (61.4–88.2)	76.7 (61.4–88.2)	76.7 (61.4–88.2)	0.98
Not acute scrub typhus	10	33					

CI = confidence interval; IgM = immunoglobulin M; PPV = positive predictive value; NPV = negative predictive value.

* Immunofluorescence assay (IFA) IgM antibody and 47-kDa real-time polymerase chain reaction (PCR).

† Five cases of dengue, one Japanese encephalitis, 10 leptospirosis, 11 malaria, and 16 undiagnosed.
